# Supplementary material for: Associations between Milk Intake and Sleep Disorders in Chinese Adults: A Cross-Sectional Study
Source: Nutrients. 2023 Sep 21;15(18):4079. doi: 10.3390/nu15184079 (PMC10536886; doi:10.3390/nu15184079)
Supplement: Supplementary file 1 [file nutrients-15-04079-s001.zip › nutrients-2567010-supplementary.pdf]

Supplementary tables and figures

Supplementary Table S1: Correlation analysis between baseline characteristics

	Milk intake	Sleep Quailty	Sleep Efficiency	Sleep Latency	Sleep Duration	Sleep Disturbances	Daytime Dysfunction	Sleep Medication	Sleep Disorders	PSQI Global Scores
**Milk intake**	1.000									
**Sleep Quailty**	-0.042	1.000								
**Sleep Efficiency**	0.016	0.300 ***	1.000							
**Sleep Latency**	-0.053	0.535 ***	0.250 ***	1.000						
**Sleep Duration**	0.045	0.266 ***	0.502 ***	0.238 ***	1.000					
**Sleep Disturbances**	-0.137 ***	0.158 ***	0.155 ***	0.170 ***	0.004	1.000				
**Daytime Dysfunction**	-0.057	0.202 ***	0.203 ***	0.145 ***	0.133 ***	0.315 ***	1.000			
**Sleep Medication**	0.013	0.290 ***	0.113 **	0.208 ***	0.100 **	0.060	0.086 *	1.000		
**Sleep Disorders**	-0.073 *	0.365 ***	0.547 ***	0.400 ***	0.478 ***	0.333 ***	0.470 ***	0.145 ***	1.000	
**PSQI Global Scores**	-0.066	0.599 ***	0.674 ***	0.603 ***	0.580 ***	0.399 ***	0.480 ***	0.261 ***	0.828 ***	1.000

Note: All data were reported as Spearman correlations. *p < 0.05, **p < 0.01, ***p < 0.001.

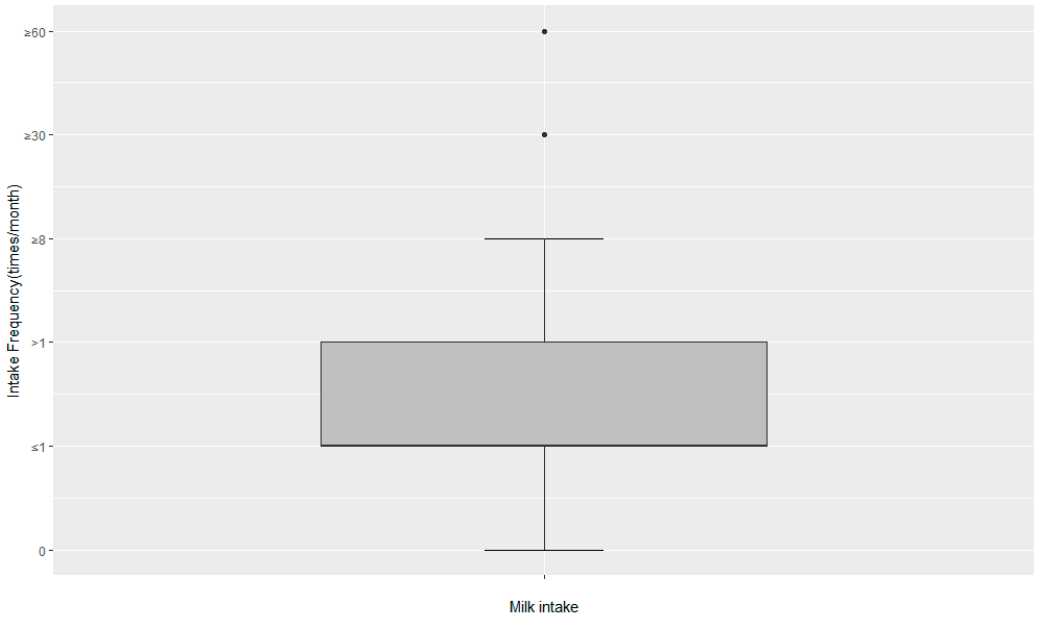

Supplementary Figure S1: Distribution of milk intake frequencies

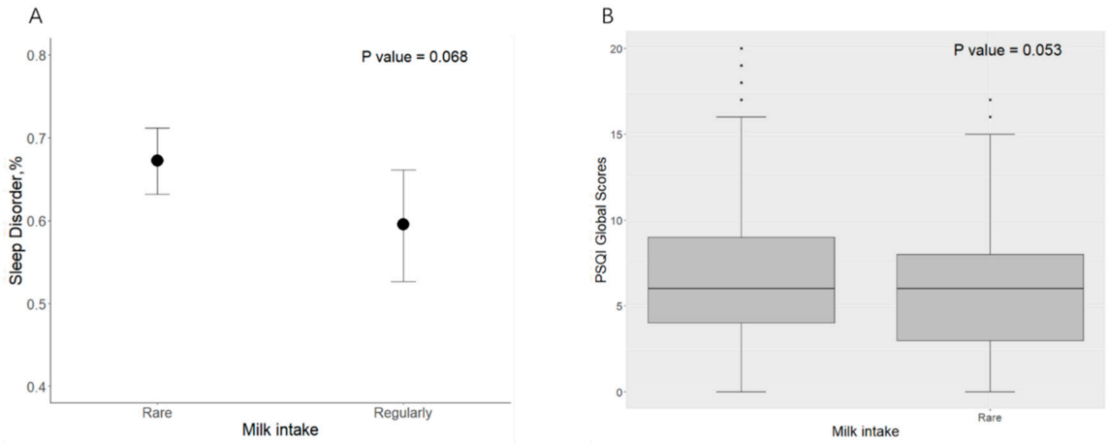

Supplementary Figure S2: The prevalence of sleep disorders in different groups of milk intake.

Note: (A) The percentage of sleep disorders and 95% CI between Rare and Regularly. (B) A third of the frequency of PSQI Global Score is between Rare and Regularly.

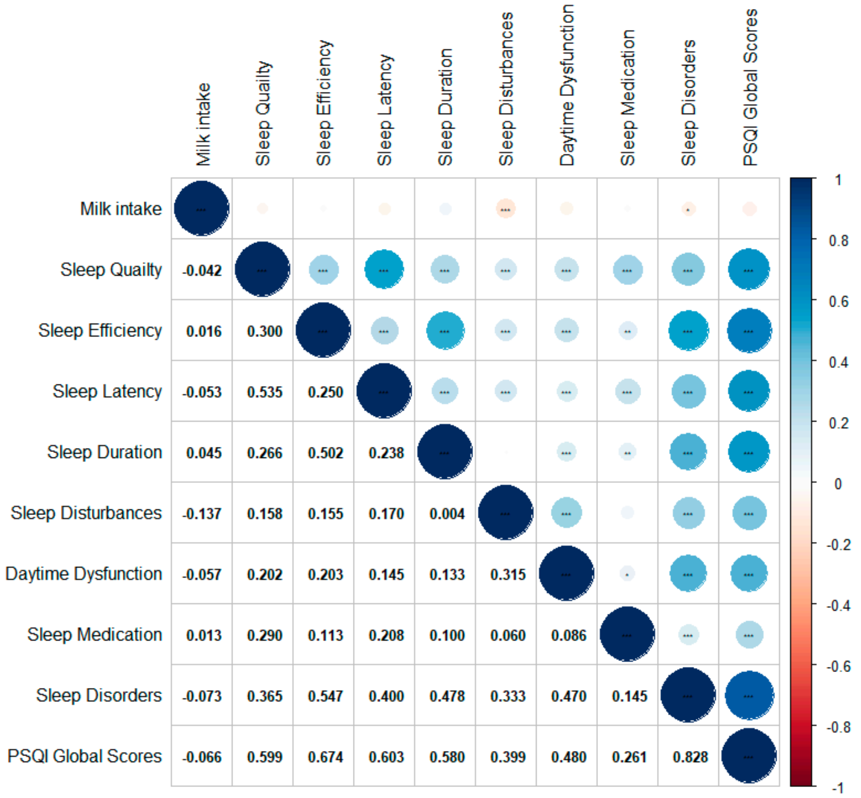

Supplementary Figure S3: Correlation analysis between baseline characteristics

Note: All data were reported as Spearman correlation analyses. *$p < 0.05$, **$p < 0.01$, ***$p < 0.001$.

Disease type	Cases/participants		Adjusted OR (95%CI)	P value
	Rare	Regularly		
Sleep Disorders	372/553	128/215	0.724 (0.511, 1.026)	0.682
PSQI Components				
Low Sleep Quality	138/553	45/215	0.842 (0.549, 1.275)	0.422
Low Sleep Efficiency	278/553	112/215	0.972 (0.686, 1.376)	0.871
Long Sleep Latency	141/553	44/215	0.845 (0.600, 1.190)	0.334
Short Sleep Duration	333/553	140/215	1.224 (0.864, 1.743)	0.259
Sleep Disturbances	520/553	184/215	0.493 (0.279, 0.874)	0.015*
Daytime Dysfunction	395/553	141/215	0.833 (0.583, 1.194)	0.316
Used Sleep Medication	20/553	9/215	1.075 (0.416, 2.600)	0.876

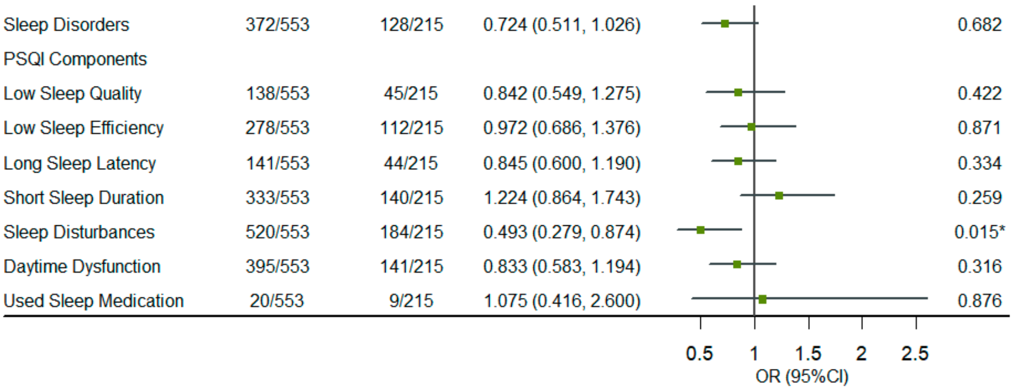

Supplementary Figure S4: Odds ratios of sleep disorders or PSQI components and corresponding 95% CIs according to tertiles of milk intake. Note: logistic regression models adjusted for age, gender, BMI, smoking, alcohol consumption, dietary confounders (salt, fruit intake, vegetable intake, seafood intake, red meat intake, egg intake, soy product intake, nuts intake, sugar-sweetened beverages intake) and clinical factors (hyper-tension, diabetes, coronary heart disease, anxiety, and depression). *p < 0.05.
